# Functional Mitochondria Are Important for the Effect of Resveratrol

**DOI:** 10.3390/molecules22050847

**Published:** 2017-05-20

**Authors:** Anne L. Widlund, Kaushal Baral, Louise T. Dalgaard, Ole Vang

**Affiliations:** Department of Science and Environment, Roskilde University, Roskilde DK-4000, Denmark; annewidlund@gmail.com (A.L.W.); kaushal.baral1@gmail.com (K.B.); ltd@ruc.dk (L.T.D.)

**Keywords:** mitochondria, resveratrol, HeLa, 143B, wild type, Rho 0, oxygen consumption rate, oxidative stress

## Abstract

Resveratrol (Resv) is a polyphenol reported to modulate mitochondrial activity. The aim was to use HeLa and 143B cells to characterize the action of Resv on mitochondrial activity, cell size and proliferation using wild type (WT) and Rho 0 cells deficient in mitochondrial DNA. In both HeLa WT and Rho 0 cells, the oxygen consumption rate (OCR) was increased at 20 µM Resv after 24 h, whereas only a non-significant increase of OCR was observed in 143B WT cells. Resv decreased cell number concentration-dependently in both WT and Rho 0 cell types. An increased cell diameter was observed in HeLa WT, but not in Rho 0 when treated with Resv. Overall, the findings presented indicate that functional mitochondria are a prerequisite for cell enlargement by Resv.

## 1. Introduction

Mitochondria are highly dynamic organelles that are involved in the regulation of cellular metabolism, redox regulation, and programmed cell death/apoptosis. Mitochondria constitute the principal energy source of the cell and convert nutrients into energy through cellular respiration [1].

Resveratrol (3,4′,5-trihydroxy-*trans*-stilbene) (Resv) is a naturally occurring polyphenol found in a high number of unrelated plant species including grapes, cranberries and peanuts, and also in a number of herbal remedies [2]. Resv shows cytostatic action, prolongs the cell cycle S phase [3], enlarges cell size [4], and there is a clear anticarcinogenic activity of Resv in animal experiments [5,6].

Previously, an increase in mitochondrial size by Resv has been observed in vivo [7,8] and, in isolated rabbit renal tubules, experiments have demonstrated increased basal respiration following Resv exposure [9]. On the other hand, experiments with isolated mitochondria show that Resv decreased the activity of several complexes in the electron transport chain (ETC) [10,11] and directly interacted with complex I [12]. Therefore, inhibition of the cellular respiration is suggested to be one of the primary targets of the cytotoxic effects of resveratrol [13].

A proper cellular metabolism is a prerequisite for cellular proliferation, differentiation and apoptosis. Impaired mitochondria may alter cellular redox homeostasis, ATP production, the biosynthetic and secretory function of cells as well as nuclear gene expression by changing retrograde signaling pathways. Resv-induced mitochondrial biogenesis would act to overcome these impairments [7,8,14]. When mitochondrial biogenesis is stimulated, as observed for Resv, a lower flow rate of electrons per unit of mitochondria is obtained, which decreases the propensity for free radical formation [15,16]. Resv protects mitochondria against oxidative stress through AMP-Activated Protein Kinase-mediated Glycogen Synthase Kinase-3β inhibition downstream of the Poly (ADP-ribose) polymerase-LKB1 pathway [17]. Since Rho 0 cells’ mitochondria are devoid of mtDNA and do not have oxidative phosphorylation, they can be used as a model for testing the molecular effects of lack of proper mitochondrial activity compared to a wild type (WT). The present experiments describe the effects of Resv on several mitochondrial activity parameters in HeLa WT and Rho0 cells. It is demonstrated that Resv treatment decreases cell numbers and proliferation, while an increase in cell size in HeLa WT is observed. Nevertheless, Resv increased OCR in both HeLa WT and Rho 0, whereas, for the cell line WT 143B and derived Rho0 cells, OCR was only altered by Resv in WT cells.

## 2. Results

### 2.1. Effect of Resveratrol on Cell Numbers, Proliferation and Diameter

**Figure 1:**
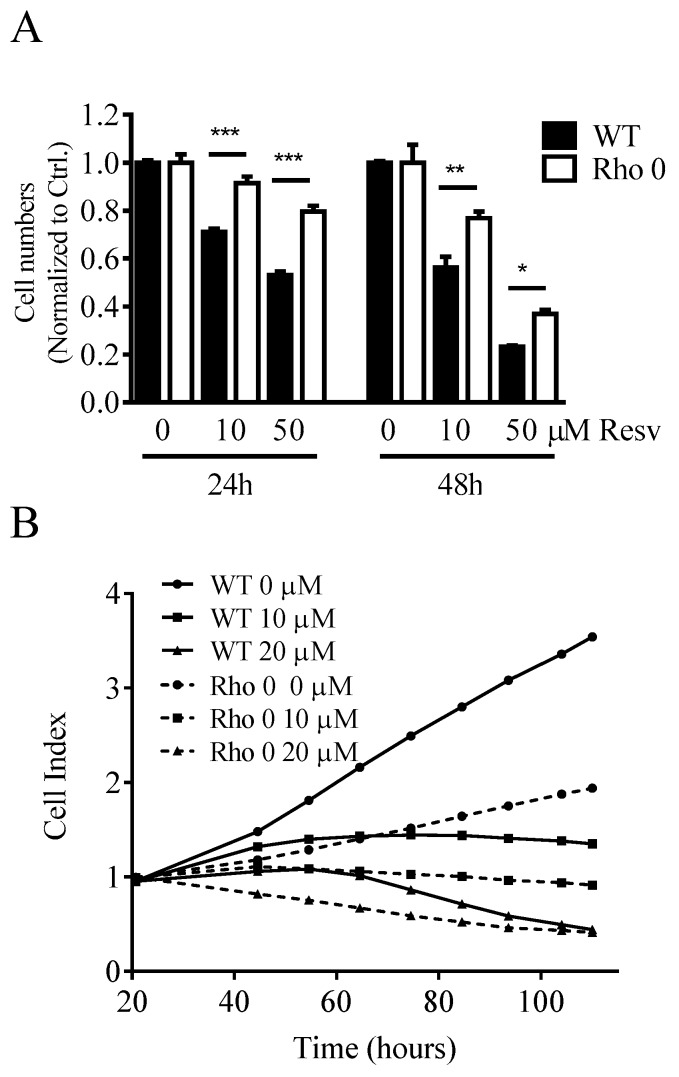

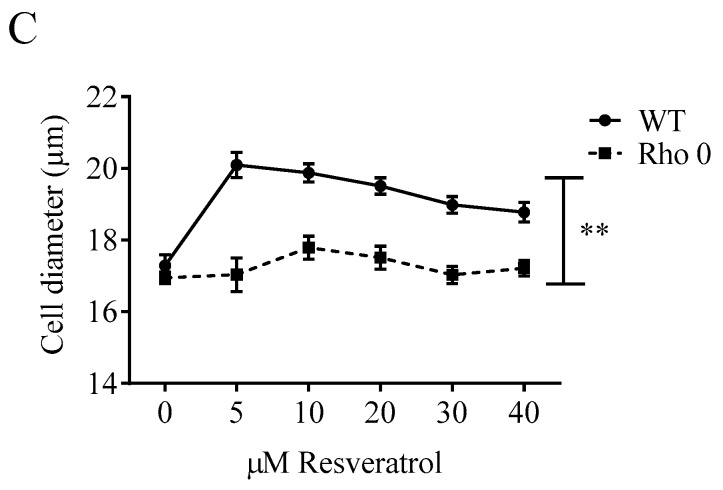
Effect of Resveratrol on cell number, proliferation and cell diameter. (**A**) normalized cell counts of HeLa wild type (WT) and HeLa Rho 0 cells treated with Resv (10 and 50 µM for 24 and 48 h). HeLa WT 10, 50 µM Resv vs. HeLa Rho 0 10, 50 µM Resv, *p* < 0.001 (***) at 24 h Cell counts in HeLa WT 10 µM Resv vs. HeLa Rho 0 10 µM Resv, *p* < 0.01 (**) and HeLa WT 50 µM Resv vs. HeLa Rho 0 50 µM Resv, *p* < 0.05 (*) at 48 h; (**B**) impedance curves of HeLa WT and Rho 0 cells treated with Resv in long-term exposure (110 h); and (**C**) cell diameter in HeLa WT and HeLa Rho 0 cells treated for 24 h with 5–40 µM Resv. HeLa WT compared with Rho 0 when treated with 5 to 40 µM Resv, *p* < 0.01(**). All values are a pool of three independent experiments with a determination of four replicates in (**A**), (**C**) and two replicates in (**B**). ANOVA/Bonferroni used for statistical analysis.

Cell proliferation was studied in both HeLa WT and Rho 0 cells by following the cell number counts up to 48 h with or without Resv treatment. Resv decreased the cell number concentration-dependently in both HeLa WT and HeLa Rho 0 (Figure 1A). The cell number counts were significantly lower in HeLa WT compared with HeLa Rho 0 at both 10 and 50 µM Resv at 24 h (*p* < 0.001 (***)) and 48 h (*p* < 0.01 (**), *p* < 0.05 (*)). As the number of cells exposed to 50 µM Resv at 24 and 48 h still is higher than the numbers at 0 h, a cytostatic effect of Resv is more likely than a cytotoxic effect. This further indicates that the absence of functional mitochondria decreases the effect of Resv on cell proliferation. However, the stronger effect of Resv in HeLa WT cells could in part be explained by a higher proliferation rate in HeLa WT relative to HeLa Rho 0 cells, also shown by the impedance data (Figure 1B). Resv increased the cell diameter in HeLa WT already at 5 µM Resv lasting up to 40 µM Resv, whereas Resv treatment did not increase cell diameter in HeLa Rho 0 (Figure 1C). The obtained data from HeLa WT and HeLa Rho 0 cells were supported by experiments using human osteosarcoma cells (143B). The 143B WT cells were more sensitive to Resv treatments (IC_50_ = 7.3 µM at 48 h) in contrast to IC_50_ = 13.0 µM, for 143B Rho 0. This indicates stronger reduction in cell number for 143B WT than for Rho 0 (Supplemental Figure S1). Therefore, functional mitochondria are a prerequisite for the cell enlargement effect of Resv. 

### 2.2. Effect of Resveratrol Exposure for 24 h on Oxygen Consumption Rate in HeLa Cells 

**Figure 2:**
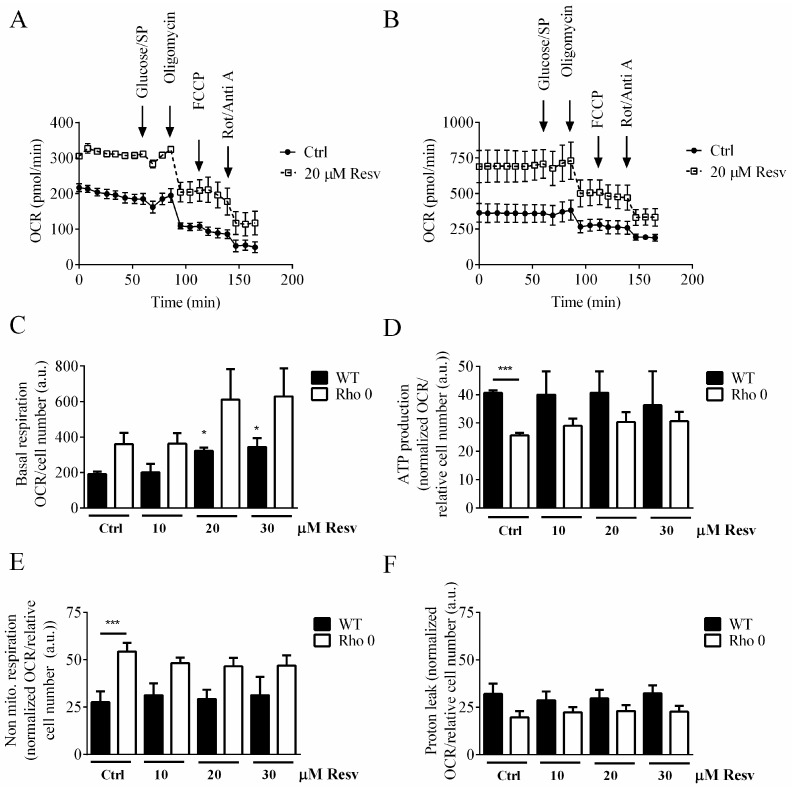
Mitochondrial activity of HeLa WT and HeLa Rho 0 following 24 h exposure to resveratrol. (**A**) Oxygen consumption rates (OCR), HeLa WT trace; (**B**) OCR, HeLa Rho 0 trace; (**C**) average of basal respiration measurements, HeLa WT and Rho 0, were HeLa WT 20 and 30 µM Resv compared to HeLa WT Ctrl (*: *p* < 0.05); (**D**) relative OCR related to ATP production of HeLa WT and Rho 0 calculated data after addition of oligomycin, were HeLa WT ctrl compared to HeLa Rho 0 Ctrl (***: *p* < 0.001); (**E**) relative rate of non-mitochondrial respiration of HeLa WT and Rho 0, calculated data after addition of rotenone/antimycin A, were HeLa WT ctrl compared to HeLa Rho 0 Ctrl (***: *p* < 0.001); (**F**) relative OCR related to proton leak of HeLa WT and Rho 0 calculated after addition of oligomycin minus non mitochondrial respiration. Data are presented as mean of three experiments ± SEM. ANOVA/Bonferroni used for statistical analysis.

To determine if Resv has an effect on mitochondrial OCR, we used the XF24 Extracellular Flux Analyzer (Agilent, Glostrup, Denmark) to characterize the effect of Resv on the electron transport chain (ETC) by applying oligomycin, carbonyl cyanide-p-(trifluoromethoxy)phenylhydrazone (FCCP) and rotenone/antimycin A. Extra glucose and sodium pyruvate were provided as additional energy sources. An overall higher OCR signal is observed in HeLa Rho 0 compared with HeLa WT when treated with Resv, which is evident from the XF24 respiration traces (Figure 2A,B) and the basal OCR (Figure 2C). 

Treatment with Resv for 24 h showed a concentration-dependent increase in basal OCR, *p* < 0.05 (*) at 20 and 30 µM Resv treatments (Figure 2C). Neither the OCR related to ATP production (Figure 2D), or to non-mitochondrial respiration (Figure 2E), nor proton leak (Figure 2F) was affected by Resv treatment. Despite optimization of FCCP levels used for the OCR assay, it was not possible to uncouple the respiration of HeLa cells, which is evident from the traces shown in Figure 2A,B.

To evaluate whether this relatively high OCR in HeLa Rho 0 cells could be specific for this cell line or a general phenomenon, a similar set of experiments was performed using 143B WT and the corresponding 143B Rho 0 cells. The OCR is increased in 143B WT compared with HeLa WT also when treated with Resv (Figure 2A and Supplemental Figure S2A). The OCR in both HeLa WT and 143B Rho 0 cell lines are similar (Figure 2B and Supplemental Figure S2A). Furthermore, the increased OCR observed for HeLa Rho 0 following exposure to Resv was not observed in 143B Rho 0 cells when tested up to 48 h Resv exposure. Non-significant changes of the various parts of the OCR are observed for 143B WT or Rho 0 cells following exposure to 5 and 10 µM Resv up to 48 h (Supplemental Figure S2C–E), even though the cell number is significantly lower when exposed to 10 µM Resv (Supplemental Figure S1).

### 2.3. Resveratrol Effect on Mitochondrial Mass, Membrane Potential and Reactive Oxygen Species in HeLa WT and Rho 0 Cells

**Figure 3:**
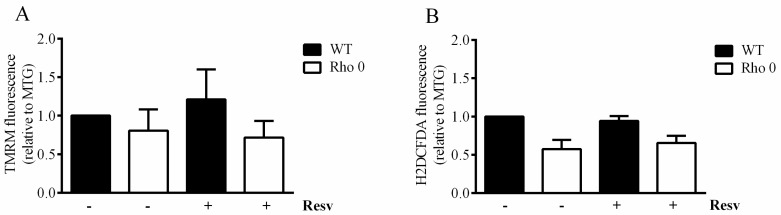
Mitochondrial membrane potential and cellular reactive oxygen species in HeLa WT and Rho 0 cells. Cells were treated with or without 20 µM Resv for 24 h, and mitochondria were labeled using (**A**) Tetramethylrhodamine, methyl ester, perchlorate (TMRM), as an estimate of mitochondrial membrane potential; (**B**) H_2_DCFDA to visualize reactive oxygen species. The estimates are shown as relative to a simultanous estimate of mitochondrial mass by Mitotracker green (MTG). Data are presented as mean ± SEM (*n* = 3) analyzed using *t*-test.

The mitochondrial membrane potential estimated as Tetramethylrhodamine methyl ester, perchlorate (TMRM) relative to Mitotracker Green (MTG), is not changed following exposure to Resv nor when comparing HeLa Rho 0 with HeLa WT (Figure 3A). Furthermore, no changes in oxidative stress were observed after Resv treatment or when comparing the oxidative stress in HeLa Rho 0 and HeLa WT cells (Figure 3B). The MTG fluorescence was not increased by Resv treatment, but a significantly increased MTG signal was observed in HeLa Rho 0 relative to HeLa WT.

### 2.4. Effect of Resveratrol Exposure for 24 h on Extracellular Acidification Rate (ECAR)

**Figure 4:**
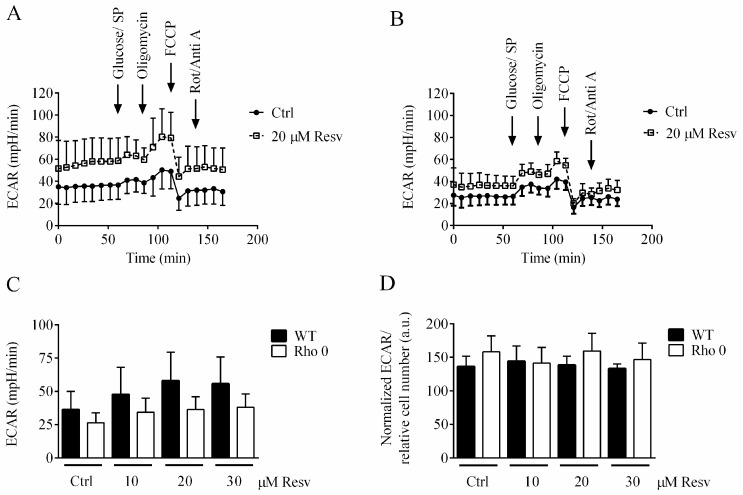
Glycolytic activity of HeLa WT and HeLa Rho 0 following 24 h exposure to resveratrol. (**A**) extracellular acidification rate (ECAR), HeLa WT trace; (**B**) ECAR, HeLa Rho 0 trace; (**C**) average of basal ECAR, HeLa WT and Rho 0; (**D**) relative ECAR of HeLa WT and Rho 0 determined after oligomycin addition. Data are presented as mean of three experiments ± SEM.

Extracellular acidification rate (ECAR) is predominantly the result of glycolytic activity. It is investigated if Resv affected ECAR in HeLa WT and HeLa Rho 0, especially based on the high OCR rates seen in the HeLa Rho 0 cells (Figure 2B). Resv concentration-dependently increased basal ECAR but had no effect on oligomycin-induced glycolysis (Figure 4). Overall, HeLa WT cells showed higher ECAR compared to HeLa Rho 0, also when treated with Resv, which is evident form the XF24 ECAR traces (Figure 4A,B) and the average of basal ECAR measurements (Figure 4C). This indicates a high reliance on glycolysis for energy production in both HeLa WT and Rho 0, even when treated with Resv.

### 2.5. Effect of Resveratrol on Mitochondria Related mRNA Transcript Levels

**Figure 5:**
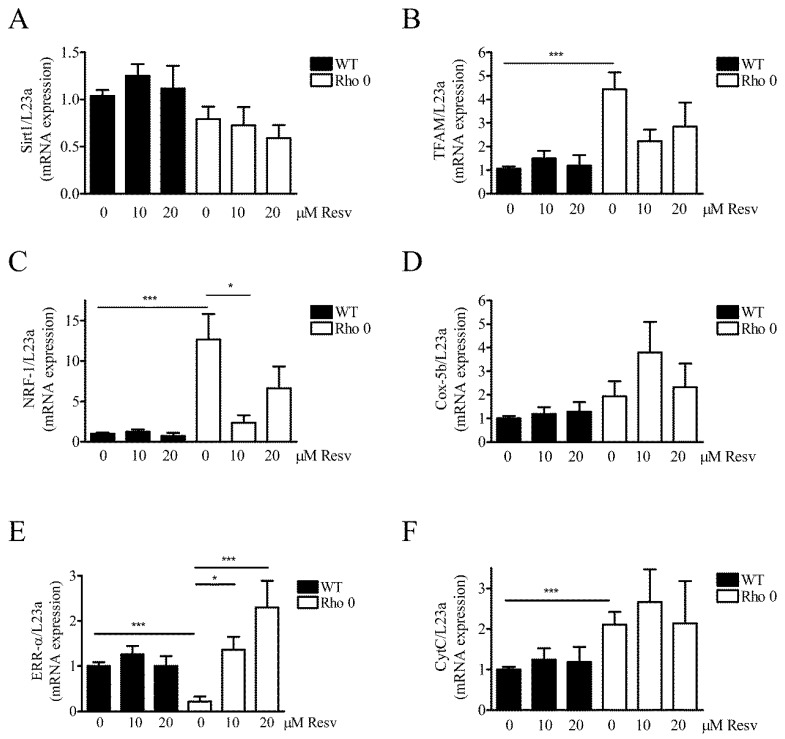

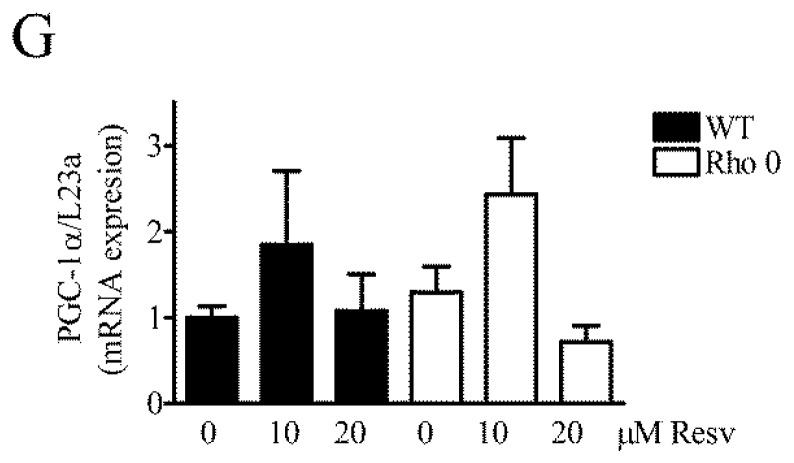
Effect of Resveratrol on expression levels of mRNA transcripts related to mitochondrial function. Relative gene expression of key genes related to mitochondrial function measured by Q-RT-PCR (quantitative reverse transcriptase polymerase chain reaction) in HeLa WT and HeLa Rho 0 cells treated with 10 and 20 µM Resv for 24 h. Expression of (**A**) NAD (nicotineamide dinucleotide)-dependent deacetylase sirtuin-1 (SIRT1); (**B**) mitochondrial transcription factor A (TFAM), HeLa WT compared to HeLa Rho 0 (***: *p* < 0.001) untreated; (**C**) nuclear respiratory factor 1 (NRF-1 also known as NFE2L1), HeLa WT compared to HeLa Rho 0 (***: *p* < 0.001) untreated and HeLa Rho 0 untreated compared to HeLa Rho 0 10 µM Resv (*: *p* < 0.05); (**D**) cytochrome C oxidase 5b (COX5b); (**E**) estrogen-related receptor alpha (ERR-α), HeLa WT compared to HeLa Rho 0 (***: *p* < 0.001) untreated and HeLa Rho 0 untreated compared to HeLa Rho 0 10 µM Resv (*: *p* < 0.05), compared to 20 µM (***: *p* < 0.001); (**F**) cytochrome complex (Cyt C), HeLa WT compared to HeLa Rho 0 (***: *p* < 0.001) untreated, (**G**) proliferator-activated receptor coactivator-1α (PGC-1α). All data are presented as relative to levels of RPL23a and are shown as mean ± SEM (*n* = 6). Statistical analysis performed using a *t*-test.

To evaluate further if HeLa WT and HeLa Rho 0´s mitochondria were affected by Resv treatment, the expression of several key mRNA transcripts related to mitochondrial function was measured (Figure 5). Since Resv improves mitochondrial function and protects against metabolic diseases by activating NAD-dependent deacetylase sirtuin-1 (SIRT1) [7], the mRNA expression level of Sirt1 was also measured. In general, expression levels of the measured transcripts are lower in HeLa WT than HeLa Rho 0, except for SIRT1 (Figure 5). In HeLa WT, Resv treatment does not change SIRT1 (Figure 5A). However, a trend for upregulation with Resv is observed for SIRT1, especially with 10 µM Resv treatments (Figure 5A). SIRT1 and peroxisome proliferator-activated receptor gamma coactivator-1α (PGC-1α) both induce genes related to oxidative phosphorylation and show trends for increase in transcript levels with Resv treatment (Figure 5A,G). Mitochondrial transcription factor A (TFAM), a nuclear encoded mitochondrial transcription factor, which is indispensable for expression of key mitochondrial encoded genes (17), is increased in HeLa Rho 0 compared to HeLa WT, *p* < 0.001 (***) (Figure 5B). Nuclear respiratory factor 1 (NRF-1, also known as NFE2L1) is expressed in low levels in HeLa WT, and >10 fold upregulated in HeLa Rho 0 (Figure 5C) (*p* < 0.001, (***)). Treatment of HeLa Rho 0 with 10 µM Resv decreased NRF1 mRNA levels (*p* < 0.05 (*)). Interestingly, Resv treatment reduced NRF1 mRNA levels of Rho 0 to HeLa WT levels. Cox5b show no changes in mRNA levels (Figure 5D). Estrogen-related receptor alpha (ERR-α) is markedly decreased in HeLa Rho 0 compared with WT (*p* < 0.001 (***)). Moreover, in HeLa Rho 0 cells, Resv concentration-dependently increased mRNA levels of ERR-α (10 µM Resv; *p* < 0.05 (*), 20 µM Resv; *p* < 0.001 (***)). Cytochrome complex (Cyt C) was increased by 2 fold in HeLa Rho 0 compared to WT, (*p* < 0.001 (***) (Figure 5F), whereas Resv had no effect on Cyt C mRNA levels. PGC-1 α transcript levels were similar in HeLa WT and Rho 0 cells and were unaffected by Resv treatment (Figure 5G).

## 3. Discussion

The mitochondrion is a central organelle for cellular proliferation and development and one of the proposed cellular targets for the effect of Resv. To analyze this in real time, a comparison of the effect of Resv on cells with and without functional mitochondria was made using the cervical cancer cell line HeLa with supporting data from 143B osteosarcoma cells. 

The decreased cell proliferation rate in HeLa Rho 0 compared to HeLa WT is consistent with less ATP being produced via oxidative phosphorylation in the mitochondria, but data also showed (1) a higher mitochondrial mass; (2) increased expression of most of the tested mitochondria related mRNAs in HeLa Rho 0; and (3) an increased basal respiration in HeLa Rho 0. These features of HeLa Rho 0 cells indicate that the increased mitochondrial mass and TFAM expression could be a result from a compensatory survival strategy. In turn, a larger amount of mitochondria, which are dysfunctional, may result in an increased oxidative stress level. This is partly observed in HeLa Rho 0 cells when calculating the absolute values but not the oxidative stress level relative to the mitochondrial mass. Absence of the mitochondria encoded subunits prevents the assembly and function of mitochondrial respiratory complexes [18]. However, HeLa Rho 0 mitochondria retain a proton gradient, which is essential for protein import into mitochondria and for counteracting apoptosis [19]. This proton gradient is generated by hydrolyzing ATP imported from the cytoplasm by the ATP-ADP shuttle. ATP is hydrolyzed by the ATP synthase working in reverse mode to generate a transmembrane proton gradient [20]. The enhanced expression of TFAM, Cyt c and most of the analyzed mitochondria related transcripts therefore indicates increased mitochondrial mass or volume in HeLa Rho 0.

The enhanced basal respiration in HeLa Rho 0 compared with HeLa WT can partially be explained by the increased non-mitochondrial respiration. In Rho 0 cells, NADH_2_ (reduced NAD^+^) is re-oxidized and electrons delivered to plasma-membrane oxidases, in order to recycle NAD^+^ for use in glycolysis [21]. This flow of electrons will provide an increased non-mitochondrial OCR as seen in Figure 2E. However, basal respiration in Rho 0 is increased above what can be explained by increased non-mitochondrial respiration, leaving some oxygen consumption unaccounted for. It should be noted, however, that substantial cell membrane oxygen consumption has been observed for different cell lines, including HelA subtypes [21]. For comparison, 143B Rho 0 cells had a lower OCR (much lower than the 143B WT counterpart), which was unaffected by Resv treatment. To gain mechanistic insight into unaccounted non-mitochondrial respiration in HeLa Rho0 cells, these should be further studied using inhibitors, since it is evident from other studies that plasma membrane redox system is a Resv target [22]. Moreover, when comparing the OCR/ECAR ratios of the Hela WT and HelA Rho 0 versus the 143B WT and 143B Rho 0, there were similar ratios for the two WT cell lines (4.8 ± 1.6) in contrast to a much higher ratio in HeLa Rho 0 cells (8.4 ± 1.0) and a much lower ratio for 143B Rho 0 cells (2.7 ± 0.9). This indicates different adaptive strategies for Rho 0 cells to obtain the necessary energy input.

Cells in general, and especially tumor cells, require a sufficient amount of ATP in order to synthesize bioactive compounds for rapid cell proliferation [23] and different tumor cells use different strategies to gain sufficient amounts of ATP for the increased cell proliferation. Whereas some have increased glycolysis, others have an enhanced oxidative phosphorylation pathway [24]. Our present data indicate that HeLa Rho 0 and WT have a different source of ATP production, due to the lack of well-functioning mitochondria, which also have an impact on regulation of glycolysis.

Numerous studies show a clear reduction in cell proliferation by Resv, which is confirmed by present data. Here, we significantly extend these findings by the observation that functional mitochondria are necessary for the cellular response to Resv and a Resv-induced cell enlargement. Previous findings show that Resv does not have an effect on cell doubling time in PC3 Rho 0 cells compared to the corresponding WT cells [25]. Cell size is coupled to cell cycle progression and is affected by both internal and external cues. Cells must reach a certain size, before they progress in the cell cycle. Here is presented that Resv increases cell size in HeLa WT but not in the mitochondria deficient Rho 0 cells. In some cell types, Resv activates different pathways e.g., mechanistic target of rapamycin (mTOR) [26], which could be the explanation to the increased cell size observed by Resv treatment in HeLa WT cells. 

Resv increases basal OCR in both HeLa cell lines, but to a greater extent in HeLa WT, suggesting that mitochondrial function is indeed increased by Resv. On the other hand, the non-mitochondrial respiration is highly increased but only in HeLa Rho 0 cells, which indicates that Resv regulates a non-mitochondrial target as well. In contrast to the increased oxygen consumption rates, Resv did not increase ROS levels (Figure 3B). Previous data reports that Resv increases the mitochondrial mass in HeLa WT cells [7,8,14,24], which this experiment was unable to replicate. 

Resv shows an increase in both mitochondrial oxidative as well as glycolytic activity for HeLa WT and Rho 0 (only significant for WT). Our data support the notion that HeLa WT has a propensity for using glycolysis for energy production, which underpins the importance of mitochondrial recycling of NADH (Figure 4C). Catabolite repression is an important part of a cell’s global control system. It is assumed that interplay of Resv with membranes of wild type cells probably can cause a negative effect on the catalytic region of ETC proteins and F_0_F_1_-ATP, which is the opposite for Rho 0 cells. It is also clear that this cell model has decreased oligomycin response and absent FCCP response (Figure 2A,B and Figure 4A,B).

Previous reports showed an inhibition of ETC by Resv using isolated mitochondria [10,11]. Such inhibition was not observed in the present experimental setup using intact cells. Even cells exposed to 10 µM Resv for 48 h showed the same OCR even though the cell number was decreased significantly, which indicates an increased ETC activity per cell.

Most of the mitochondria related mRNAs levels, except Sirt1 and PGC-1α, are increased in HeLa Rho 0 cells. Thus, there appears to be nuclear compensatory transcription in response to the dysfunctional mitochondria. The reference gene used for normalization showed similar Ct values between cell lines. Resv only has a very minor impact on the level of these mRNAs, and if so, at a lower concentration of Resv than the concentration which increases basal OCR (Figure 2C). The minor effect of Resv on mRNA levels in treated HeLa WT could indicate that cellular modulation by Resv primarily acts at protein level [26].

HeLa Rho 0 show more than expected oligomycin linked reduction of OCR. Both HeLa Rho 0 and WT do need a functional oligomycin-sensitive ATP synthase as discussed above, but it is more likely that the HeLa Rho 0 cells used may not have a completely malfunctioning electron transporting chain. Therefore, in order to confirm the current findings in HeLa cells, additional Rho 0 cell types should be investigated.

The described experiments showed cellular effects of Resv in the range of 5 to 50 µM after 24 h. According to Yang et al., more than 90% of the administrated Resv is degraded after 24 h in sodium bicarbonate containing media, and the actual concentrations of Resv are assumed to be lower than the initial dose [27]. As Resv binds to proteins and lipid structures in the media, the high degree of degradation described by Yang et al. is likely an overestimation. 

The partly lacking effect of Resv in HeLa Rho 0 could be explained by the fact that Resv inhibits ATP synthase. Recent experiments have shown that mitochondrial calcium overload and the apposite is triggered in HeLa cells and other cancer cell types [28], which support the notion; functional mitochondria are important for the effect of Resv.

## 4. Materials and Methods

### 4.1. Materials

HeLa WT and Rho 0 derived from cervical cancer cells were a gift from Dr. Claus Desler, Department of Cellular and Molecular Medicine, University of Copenhagen, Denmark [29]. Dulbecco’s modified Eagle’s medium (DMEM), Hank's Balanced Salt Solution (HBSS), Penicillin/streptomycin and uridine were obtained from Sigma-Aldrich (Broendby, Denmark). Fetal bovine serum (FBS) was from Merck Millipore (Hellerup, Denmark). Resveratrol was acquired from Fluxome Science (Stenloese, Denmark)—now Evolva (Reinach, Switzerland)—and dissolved in DMSO.

### 4.2. Cell Culture

HeLa WT and HeLa Rho 0 were grown in DMEM supplemented with 10% fetal bovine serum and 1% Penicillin/Streptomycin. HeLa Rho 0 medium was supplemented with 200 µM uridine. Cells were cultured in Thermo Fisher Scientific NuncTM microplates (Roskilde, Denmark).

### 4.3. Determination of Cell Number and Size 

To determine cell number and diameter in HeLa WT and HeLa Rho 0, a Beckman Coulter, Z2 Coulter particle counter and size analyzer (Pasadena, CA, USA) was used [30]. Particles with diameter size > 7 μm were included for data analysis. All cell counts were carried out in quadruplicate for each treatment with three independent experiments. The cells were treated with DMSO (as solvent) and 10–50 µM Resv for 24 and 48 h.

### 4.4. Impedance Readings

Evaluating the effects of Resv on cell proliferation was based on real-time cell-based electronic sensor technology iCELLigence System [31] (ACEA Biosciences, Inv., San Diego, CA, USA). Measurements were performed according to manufacturer´s protocol. Briefly: in an E-plate L8 dish, each well was filled with 200 µL medium and placed in the iCELLigence station in a 37 °C incubator. Before seeding the cells into the E-Plate L8 (15.000 cells per well), a background measurement was done with media. The HeLa WT and HeLa Rho 0 were cultured in E-plates L8 in duplicates for 24 h before treatment with Resv was initiated. The impedance signal is expressed as the cell index at each time point per concentration of Resv. The cells were treated with DMSO (as solvent), 10 or 20 µM Resv for up to 110 h.

### 4.5. Respirometry in Intact Cells 

The oxygen consumption rate (OCR) and extracellular acidification rate (ECAR) measurements were made using Seahorse Bioscience XF-24 instrument (Seahorse Bioscience, Copenhagen, Denmark). HeLa WT and HeLa Rho 0 were grown to 80–90% confluence and then treated with 0, 10, 20 or 30 µM Resv 24 h prior to the day of the respirometry. On the day of the assay, the cell medium was removed from the well and running medium (XF-DMEM assay buffer, Seahorse Bioscience) was placed into the wells and warmed to 37 °C for at least 30 min without 5% CO_2_ atmosphere. The injection ports of the sensor cartridge were filled with Glucose/Sodium Pyruvate (25 mM/1 mM), Oligomycin (10 µM), FCCP (30 µM), and rotenone/antimycin A (20 µM for both) (Seahorse Bioscience). The measurement cycle consisted of a 3-min mix, a 3-min wait and a 3-min measurement. Eight basal rate measurements were followed by injections, and each injection was followed by three to four measurement cycles. Activity estimates for any given treatment were based on the rates from three wells to calculate the investigated parameters. Basal OCR represents the net sum of all processes in the cell capable of consuming O_2_ including mitochondria and other oxidases. Basal ECAR is established with the first measurements, as well as OCR. Non-mitochondrial respiration is estimated by average of OCR measurements after addition of antimycin A and rotenone. Proton leak is calculated as the first measurement after addition of oligomycin subtracted from non-mitochondrial respiration. The OCR-related ATP production is calculated as proton leak subtracted from basal OCR. Basal ECAR represent the net sum of predominantly measure of lactic acid formed during glycolytic energy metabolism. After addition of oligomycin, increased glycolytic rate in response to loss of mitochondrial related ATP production is calculated as the average of oligomycin stimulated rate of ECAR.

### 4.6. Detection of Mitochondrial Mass, Mitochondrial Membrane Potential and Oxidative Stress by Flow Cytometry

After 24 h of treatment with 0 or 20 µM Resv., HeLa WT and HeLa Rho 0 cells were detached and re-suspended in HBSS buffer (10 mM HEPES, 140 mM NaCl and 25 mM CaCl2, pH 7.4). Cells were incubated with Mitotracker green (MTG) (Invitrogen M7514, Thermo Fisher Scientific, Roskilde, Denmark) (0.01 µM), Tetramethylrhodamine methyl ester (TMRM) (Invitrogen T668) (0.01 µM) at room temperature (RT) or 2’,7’-dichlorodihydrofluorescein diacetate (H2DCFDA) (Invitrogen C399) (0.1 µM) at 37 °C, for 30 min. Analysis was performed using flow cytometry (FACS Calibur, Becton-Dickinson, NJ, USA) and analyzed by BD CellQuest™ Pro Analysis software (version 4.0.2, Becton Dickinson, Khs. Lyngby, Denmark). Data were normalized to the HeLa WT solvent condition due to variation in absolute signal strength between experiments.

### 4.7. Quantitative Real Time RT-PCR Assay

Total RNA was extracted using TRI-Reagent (Sigma-Aldrich, Broendby, Denmark) according to manufacturer’s instructions. Concentration and purity of RNA were determined by NanoDrop spectrophotometer ND 1000 (Fisher Scientific, Roskilde, Denmark) and stored at −80 °C until cDNA synthesis. In addition, 1 µg of total RNA was reverse transcribed using a Superscript III Reverse transcription (Invitrogen) according to manufacturer’s instructions. The cDNA was subjected to real time PCR using QuantiTect SYBR^®^ Green PCR Kit (Qiagen, Venlo, Netherlands). A reaction contained 500 nM of each forward and reverses primer, and 2 µL of 10 fold diluted cDNA with final reaction volume 10 µL. The reaction was initiated by 95 °C for 15 min. and 40 amplification cycles were carried out with 10 s denaturation at 95 °C and 60 s annealing/elongation at 60 °C. Subsequently, a melting curve was carried out. Gene expression was normalized to mean of RPL23a rRNA signals. Oligonucleotide sequences are available on request.

### 4.8. Statistical Analysis 

All data are expressed as mean ± SEM and data are presented as a pool of replicate measurements from 2–4 independent experiments. Multiple groups were analyzed using one-way ANOVA, with Bonferroni post-test correction for multiple comparisons using GraphPad Prism (version 6.07, GrapPad, La Yolla, CA, USA). A *t*-test was used to analyze differences in mRNA levels. A probability of less than 0.05 was considered to indicate a significant difference.

## 5. Conclusions

In conclusion, functional mitochondria are a prerequisite for Resv to have an effect on cell size and in part for the modulation of cell proliferation. Presented data also show that Resv increases oxygen consumption rates as well as glycolysis in HeLa cells. Functional mitochondria are important for the effect of resveratrol.

## Supplementary Materials

The following are available online. Figure S1: Effect of Resveratrol on cell number of 143B cells (WT and Rho 0); Figure S2: Mitochondrial activity of 143B WT and 143 Rho 0 following exposure for 48 h to resveratrol.
