# Supplementary material for: Functional Mitochondria Are Important for the Effect of Resveratrol
Source: Molecules. 2017 May 20;22(5):847. doi: 10.3390/molecules22050847 (PMC6153999; doi:10.3390/molecules22050847)
Supplement: Supplementary file 1 [file molecules-22-00847-s001.pdf]

Functional mitochondria are important for the effect of resveratrol

Anne L. Widlund, Kaushal Baral, Louise T. Dalgaard and Ole Vang

Supplemental data

Materials and Methods

143B WT and Rho 0 derived from osteosarcoma cancer cells were a gift from Claus Desler, Department of Cellular and Molecular Medicine, University of Copenhagen, Denmark. The 143B cells were grown in Dulbecco's modified Eagle's medium (DMEM) from Lonza. The remaining materials and the equipment used were similar to what is described in the Material and Methods section.

Respirometry in intact cells

The oxygen consumption measurement as described in the Material and Method section, but without addition of glucose and sodium pyruvate.

The protein level in the cells analyzed in the XF24 was estimated by staining with 0.05% coomassie brilliant blue solution in a solution of 30% methanol and 10% acetic acid. After wash and solubilization with 1% SDS, the color was estimated with the absorbance estimate at 620 nm.

Results

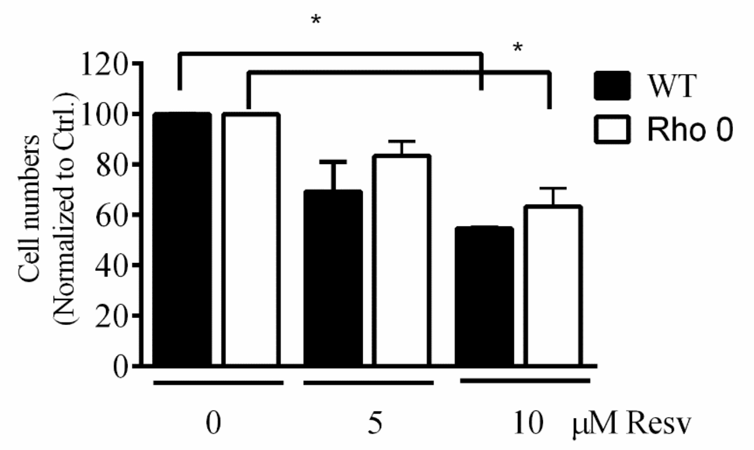

Figure S1. Effect of Resveratrol on cell number of 143B cells WT and Rho 0. Cell counts of 143B WT and 143B Rho 0 cells treated with Resv (5 and 10 μM for 48 h). 10 μM Resv vs 0 μM Resv, $p<0.05$ (*) in both cell lines. Data are presented as mean of two experiments ± range. ANOVA/Bonferroni used for statistical analyzing.

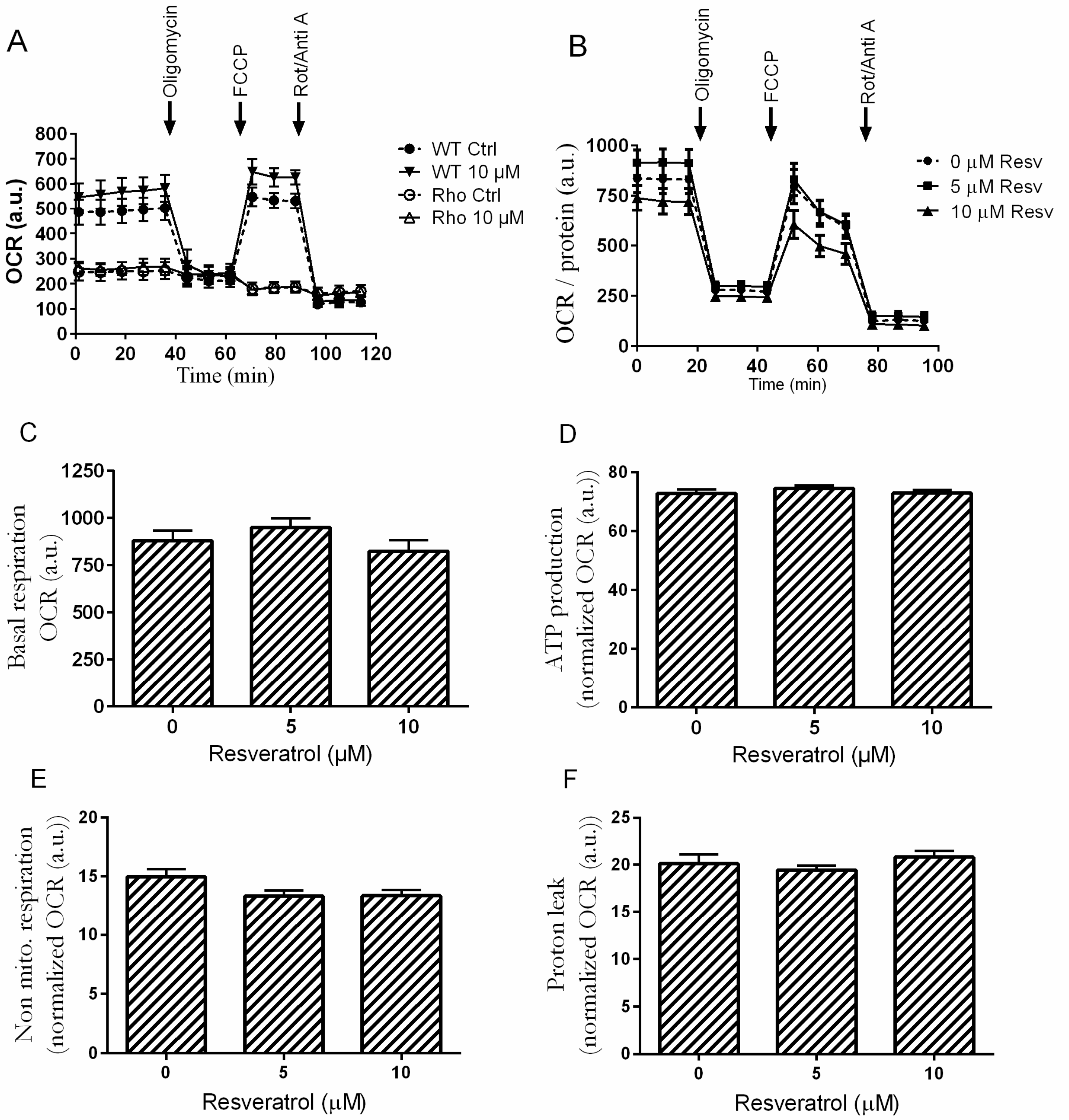

Figure S2. Mitochondrial activity of 143B WT and 143 Rho 0 following 48 h exposure to resveratrol. (A) Oxygen consumption (OCR), comparing 143B WT and 143B Rho 0 traces. (B) OCR trace of143B WT teated with 0, 5 or 10 μM Resv. (C) Average of basal respiration measurements (OCR) for 143B WT. (D) Relative OCR related to ATP production of 143B WT calculated data after addition of oligomycin. (E) Relative rate of non-mitochondrial respiration of 143B WT, calculated after addition of rotenone/antimycin A. (F) Relative OCR related to proton leak of 143B WT, calculated after addition of oligomycin minus non mitochondrial respiration. Data are presented as mean of three experiments ± SEM. ANOVA/Bonferroni used for statistical analyzing.
